# Comparison of Indirect and Direct Laryngoscopes in Pediatric Patients with a Difficult Airway: A Systematic Review and Meta-Analysis

**DOI:** 10.3390/children11010060

**Published:** 2023-12-31

**Authors:** Risa Takeuchi, Hiroshi Hoshijima, Takahiro Mihara, Shinichi Kokubu, Aiji Sato (Boku), Takumi Nagumo, Tsutomu Mieda, Toshiya Shiga, Kentaro Mizuta

**Affiliations:** 1 Bunkoukai Special Needs Center, 2765-5 Ujiie, Sakura 329-1311, Tochigi, Japan; rtake0205@gmail.com (R.T.); kentaro.mizuta.e6@tohoku.ac.jp (K.M.); 2 Division of Dento-Oral Anesthesiology, Graduate School of Dentistry, Tohoku University, 4-1 Seiryomachi, Aoba, Sendai 980-8575, Miyagi, Japan; 3 Department of Health Data Science, Graduate School of Data Science, Yokohama City University, Yokohama 236-0004, Kanagawa, Japan; mihara_t@yokohama-cu.ac.jp; 4 Department of Anesthesiology, Dokkyo Medical University, 880 Kitakobayashi, Mibu, Shimotsugagun 321-0293, Tochigi, Japan; s-kokubu@dokkyomed.ac.jp; 5 Department of Anesthesiology, School of Dentistry, Aichi Gakuin University, 2-11 Suemori-dori, Chikusa-ku, Nagoya 465-8651, Aichi, Japan; bokuaiji@dpc.agu.ac.jp; 6 Department of Anesthesiology, Saitama Medical University Hospital, Irumagun 350-0495, Saitama, Japan; tnagumo@saitama-med.ac.jp (T.N.); tmieda@saitama-med.ac.jp (T.M.); 7 Department of Anesthesiology and Pain Medicine, International University of Health and Welfare Ichikawa Hospital, 6-1-4 Kounodai, Ichikawa 272-0827, Chiba, Japan; shiga@iuhw.ac.jp

**Keywords:** indirect laryngoscopes, direct laryngoscopes, pediatric, difficult airway, meta-analysis

## Abstract

This meta-analysis was performed to determine whether an indirect laryngoscope is more advantageous than a direct laryngoscope for tracheal intubation in the setting of a difficult pediatric airway. Data on the intubation failure and intubation time during tracheal intubation were extracted from prospective and retrospective studies identified through a comprehensive literature search. Data from 10 individual articles (11 trials) were combined, and a DerSimonian and Laird random-effects model was used to calculate either the pooled relative risk (RR) or the weighted mean difference (WMD) and the corresponding 95% confidence interval (CI). Meta-analysis of the 10 articles indicated that the intubation failure of tracheal intubation with an indirect laryngoscope was not significantly different from that of a direct laryngoscope in patients with a difficult airway (RR 0.86, 95% CI 0.51–1.46; *p* = 0.59; Cochrane’s Q = 50.5; I^2^ = 82%). Intubation time with an indirect laryngoscope was also similar to that with a direct laryngoscope (WMD 4.06 s; 95% CI −1.18–9.30; *p* = 0.13; Cochrane’s Q 39.8; I^2^ = 85%). In conclusion, indirect laryngoscopes had the same intubation failure and intubation time as direct laryngoscopes in pediatric patients with a difficult airway. Currently, the benefits of indirect laryngoscopes have not been observed in the setting of a difficult pediatric airway.

## 1. Introduction

Pediatric patients are anatomically smaller than adults, and the narrowness of their oral cavity, small glottis, and large tongue make tracheal intubation difficult [1]. Furthermore, hypoxia is likely to occur because of immature lung growth, and anesthesiologists are required to perform rapid tracheal intubation [2].

Previous studies have reported that intubation is difficult in 0.04% of pediatric patients and that difficult intubation should be avoided in pediatrics because of the potentially life-threatening complications [3,4]. The indirect laryngoscope is expected to be more advantageous than the conventional direct laryngoscope for tracheal intubation because a camera is attached to the tip of the blade and the view is displayed on an external monitor. It has been reported that the tracheal intubation success rate is higher with an indirect laryngoscope than with a direct laryngoscope in pediatric patients with a difficult airway [5]. In addition, Orozco et al. reported that when Airtraq and Macintosh laryngoscope were used in pediatric patients; Airtraq significantly increased the success rate and shortened the intubation time [6]. However, there are also reports indicating that the probability of intubation is better with a direct laryngoscope than with an indirect laryngoscope [7,8]. A previous report comparing the Glidescope and direct laryngoscope in 70 neonates found that the Glidescope was not significantly different from the direct laryngoscope in terms of intubation time and glottic visualization [9]. Additionally, White et al. reported that Airtraq significantly prolonged intubation time compared to direct laryngoscopes [10]. Furthermore, a previous network meta-analysis comparing indirect and direct laryngoscopes in pediatric patients reported that there were no significant differences between the two laryngoscopes in terms of success rate, intubation time, and glottic visualization in tracheal intubation [11,12]. However, this report did not focus on patients with difficult airways, so the usefulness of the indirect laryngoscope in pediatric patients with difficult intubation is not known. Therefore, we performed a meta-analysis to determine whether indirect laryngoscopes are more advantageous than direct laryngoscopes for tracheal intubation in pediatric patients with a difficult airway.

## 2. Methods

This meta-analysis was performed in accordance with the guidelines of the Cochrane Handbook for Systematic Reviews of Interventions [13], and the manuscript was prepared in accordance with the recommendations of the Statement on Preferred Reporting Items for Systematic Reviews and Meta-Analyses [14]. The methods used for the analysis and inclusion and exclusion criteria were planned before starting the meta-analysis, and the study protocol is registered in PROSPERO (CRD42021260230). In this meta-analysis, we performed a comprehensive literature search using three electronic databases: PubMed, Cochrane Central Register of Controlled Trials, and Scopus. The following strategy, which combined free text and Medical Subject Headings (MeSH) terms, was devised for the PubMed search: (“paediatrics” [All Fields] OR “pediatrics” [MeSH Terms] OR “pediatrics” [All Fields] OR “paediatric” [All Fields] OR “pediatric” [All Fields]) AND (“difficult” [All Fields] OR “difficulties” [All Fields]) AND (“airway” [All Fields] OR “airway s” [All Fields] OR “airways” [All Fields]) AND (“laryngoscope s” [All Fields] OR “laryngoscopes” [MeSH Terms] OR “laryngoscopes” [All Fields] OR “laryngoscope” [All Fields] OR “laryngoscopic” [All Fields] OR “laryngoscopical” [All Fields] OR “laryngoscopically” [All Fields]). This study further selected additional literature through a manual search of the references of the selected studies. There were no language restrictions on the included studies. The most recent search was performed in October 2022.

The following inclusion criteria were applied: pediatric study population; difficult airway; and comparison of an indirect laryngoscope with a direct laryngoscope. Studies in patients with a normal airway, studies in manikins, and studies in which tracheal intubation was performed during cardiopulmonary resuscitation were excluded.

The selection of articles to be included in the study was independently assessed by HH and TS, and any disagreements between the two authors regarding the assignment were resolved through discussion. When discrepancies in the data were suspected, the authors of the published study were contacted directly for clarification. Working independently, the two authors extracted data on intubation failure and intubation time from eligible studies using standardized data collection forms. We calculated the rate of intubation failure because many studies had intubation success rates over 50%, leading to inconsistent weighting. (Intubation failure = Total number of patients − patients successfully intubated). Difficult airway management was defined according to the definition used in the selected studies. We also compared the adverse event rate during tracheal intubation (desaturation, oropharyngeal injury, hoarseness, laryngospasm, and esophageal intubation) between the two types of laryngoscope. The PICO (Patient/Problem/Population, Intervention/Exposure, Comparison, and Outcomes) format was applied as follows: Population, pediatric patients requiring tracheal intubation for difficult airway settings; Intervention, tracheal intubation using an indirect laryngoscope; Comparison, tracheal intubation using a direct laryngoscope; and Outcomes, intubation failure, intubation time, and adverse event rate during tracheal intubation (desaturation, oropharyngeal injury, hoarseness, laryngospasm, and esophageal intubation). Additionally, we performed subgroup analyses for indirect laryngoscope types and pediatric ages. Regarding the type of indirect laryngoscope, each study used a different indirect laryngoscope, so we analyzed each indirect laryngoscope type separately. Regarding the age of pediatrics, we analyzed intubation failure and intubation time for pediatrics under 2 or fewer years of age. Moreover, prospective and retrospective studies were analyzed separately, as subgroup analyses.

### 2.1. Risk of Bias and Assessment of the Quality of Evidence

We assessed the risk of bias and quality of evidence using the Cochrane Handbook [6] (Supplement S1) and the Grading of Recommendations Assessment, Development, and Evaluation (GRADE) [8] (Supplement S2).

### 2.2. Statistical Analysis

Statistical analyses in this study were performed using Review Manager (ver. 5.2, Nordic Cochrane Centre, The Cochrane Collaboration, Copenhagen, Denmark). In this study, data from individual trials were combined using DerSimonian and Laird random effects models. Intubation failure and side effects (desaturation, oropharyngeal injury, hoarseness, laryngospasm, and esophageal intubation) data were treated as dichotomous variables, and these pooled-effect estimates were expressed as relative risk (RR) with 95% CI. The pooled difference in intubation time between indirect and direct laryngoscopy was expressed as a weighted mean difference (WMD) with 95% CI. The homogeneity of effect size across trials was tested using the Cochrane Q statistic and the I^2^ statistic [15].

In this study, standard errors and RR values were used to create funnel plots to assess publication bias [16]. We also assessed the symmetry of the resulting plots using Begg’s test [17]. (Publication bias exists if *p*-value < 0.1).

## 3. Results

**Figure 1:**
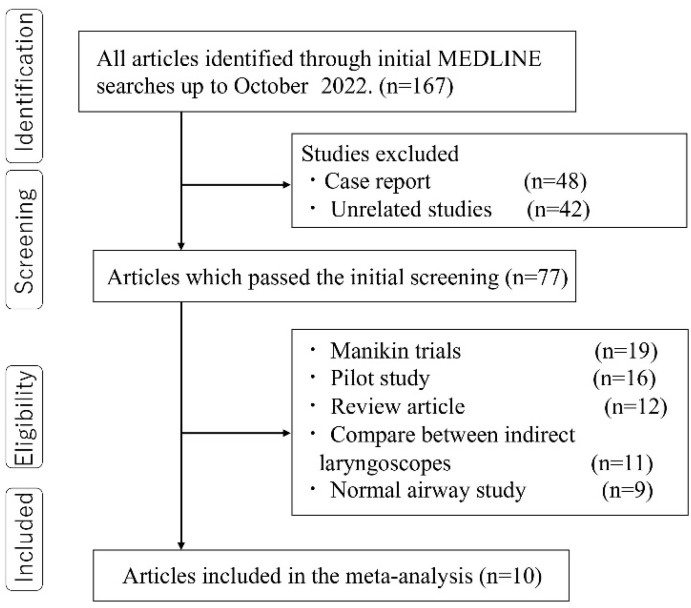
Meta-analysis flow chart. RCT, randomized controlled trial.

We first attempted to search electronic databases. This search identified 167 articles that were potentially articles for review. Among these selected studies, 90 studies were excluded because they were case reports or studies irrelevant to this meta-analysis. Of the remaining 77 articles, 67 studies were excluded for Manikin studies (n = 19), pilot studies (n = 16), review articles (n = 12), comparisons of indirect laryngoscopy alone (n = 11), or studies in patients with normal airway conditions in preoperative. Finally, 10 articles that included 11 trials met the inclusion criteria (Figure 1) [5,7,8,18,19,20,21,22,23,24]. Table 1 summarizes the details of the studies selected for this meta-analysis.

This meta-analysis included studies from 2010 to 2022. Of the 10 articles, 6 were randomized controlled trials, 3 were observational studies, and 1 was a prospective study. The C-MAC was the most frequently used indirect laryngoscope in five studies, the McGrath and Glidescope were used in two studies each, and the Airway Scope and Bullard were used in one study each. The pediatric patients participating in this study ranged in age from 0 to 18 years old. Only four studies included pediatrics under 2 years of age. Three other studies involved patients up to 18 years of age, and two studies included patients up to 10 years of age. The main intubation difficulties encountered were needed for manual in-line stabilization (two studies) and intubation in the emergency department (three studies). Other intubation difficulties included torticollis, cleft lip and palate, lateral positioning, and predicted difficult intubation (Table 1).

### 3.1. Intubation Performance

**Figure 2:**
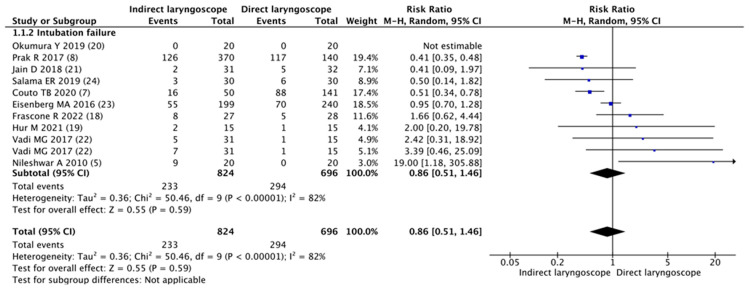
Forest plot of intubation failure for tracheal intubation using the indirect laryngoscope compared with the direct laryngoscope [5,7,8,18,19,20,21,22,23,24].

**Figure 3:**
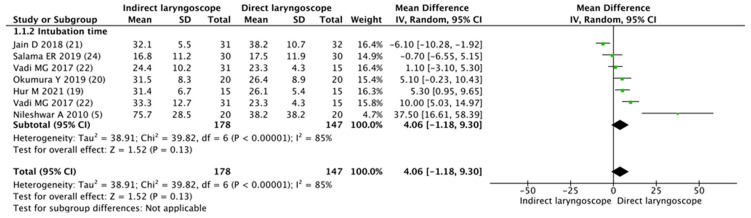
Forest plot of intubation time for tracheal intubation using the indirect laryngoscope compared with the direct laryngoscope [5,19,20,21,22,24].

Meta-analysis of the 11 trials found no significant difference in the intubation failure of tracheal intubation between the indirect laryngoscope and the direct laryngoscope in the setting of a difficult pediatric airway (RR 0.86, 95% CI 0.51–1.46; *p* = 0.59; Cochrane’s Q = 50.5; I^2^ = 82%; Figure 2). Intubation time with an indirect laryngoscope was similar to that with a direct laryngoscope (WMD 4.06 s; 95% CI −1.18–9.30; *p* = 0.13; Cochrane’s Q = 39.8; I^2^ = 85%; Figure 3).

Additionally, we performed subgroup analyses for the type of indirect laryngoscope, pediatric age, and study method (prospective or retrospective studies). Regarding the type of indirect laryngoscope, only the Bullard laryngoscope had a significantly superior s intubation failure than the direct laryngoscope (RR 19.0, 95% CI 1.18–305.9; *p* = 0.04; Cochrane’s Q = N/A; I^2^ = N/A; Supplement S3). Other indirect laryngoscopes did not differ from direct laryngoscopes in intubation failure for tracheal intubation. In terms of intubation time, the McGrath and Bullard laryngoscope significantly prolonged intubation time compared to the direct laryngoscope (McGrath; WMD 5.30 s; 95% CI 0.95–9.65; *p* = 0.02; Cochrane’s Q = N/A; I^2^ = N/A, Bullard; WMD 35.7 s; 95% CI 16.6–58.3; *p* = 0.0004; Cochrane’s Q = N/A; I^2^ = N/A; Supplement S4). We also analyzed intubation failure and intubation time for pediatrics 2 or fewer years of age. In pediatrics 2 or fewer years of age, there were no significant differences between indirect and direct laryngoscopy in both intubation failure and intubation time (Supplements S5 and S6). A subgroup analysis found no significant difference in the intubation failure between the indirect and the direct laryngoscope according to whether the study was prospective (RR 1.08, 95% CI 0.45–2.58; *p* = 0.86; Cochrane’s Q = 13.9; I^2^ = 57%) or retrospective (RR 0.79; 95% CI 0.35–1.75; *p* = 0.55; Cochrane’s Q = 33.2; I^2^ = 94%; Supplement S7). It was not possible to perform this subgroup analysis for intubation time because only prospective studies could be included.

### 3.2. Adverse Events

**Figure 4:**
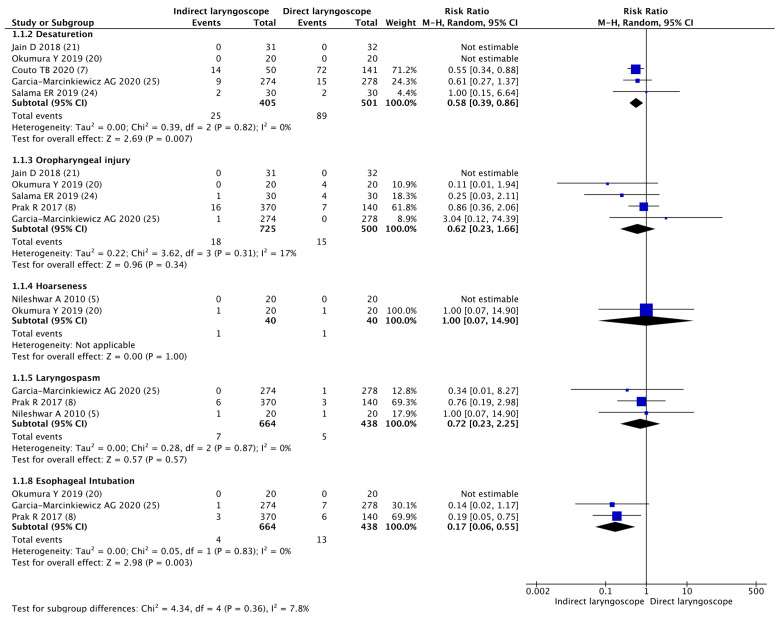
Forest plot of adverse event for tracheal intubation using the indirect laryngoscope compared with the direct laryngoscope [5,7,8,20,21,24,25].

Information on five types of adverse events (desaturation, oropharyngeal injury, hoarseness, laryngospasm, and esophageal intubation) was extracted. Compared with the direct laryngoscope, the indirect laryngoscope was associated with fewer desaturation events (RR 0.58, 95% CI 0.39–0.86; *p* = 0.007; Cochrane’s Q = 0.39; I^2^ = 0.00%) and esophageal intubation events (RR 0.17; 95% CI 0.06–0.55; *p* = 0.003; Cochrane’s Q = 0.05; I^2^ = 0.00%). There was no significant difference in the incidence of oropharyngeal injury (RR 0.62, 95% CI 0.23–1.66; *p* = 0.34; Cochrane’s Q = 3.26; I^2^ = 17%), hoarseness (RR 1.00, 95% CI 0.07–14.9; *p* = 1.00; Cochrane’s Q= N/A; I^2^= N/A), or laryngospasm (RR 0.72, 95% CI 0.23–2.25; *p* = 0.57; Cochrane’s Q = 0.28; I^2^ = 0.00%) between the indirect laryngoscope and the direct laryngoscope (Figure 4).

### 3.3. Results of GRADE Assessment

**Figure 5:**
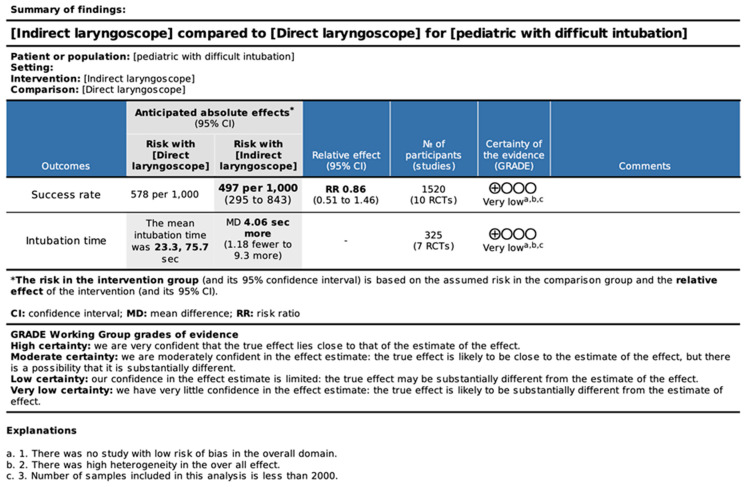
The Grading of Recommendations Assessment, Development, and Evaluation (GRADE) approach.

The GRADE assessment was very low for both intubation failure and intubation time. The risk of bias for intubation failure was high because blinding was not possible for the intubation device, heterogeneity was large, and the number of samples was small. Therefore, the GRADE evaluation was downgraded to “very low”. The risk of bias was also low for intubation time because the heterogeneity was large and the number of samples was small. The GRADE evaluation was downgraded to “very low” (Figure 5).

### 3.4. Results of Publication Bias

Begg’s test revealed an absence of major publication bias in intubation failure (Kendall’s statistic −1.00; Z value 0.02; *p* = 0.5) and intubation time (Kendall’s statistic 9.00; Z value 1.35; *p* = 0.11).

### 3.5. Results of Risk of Bias

**Figure 6:**
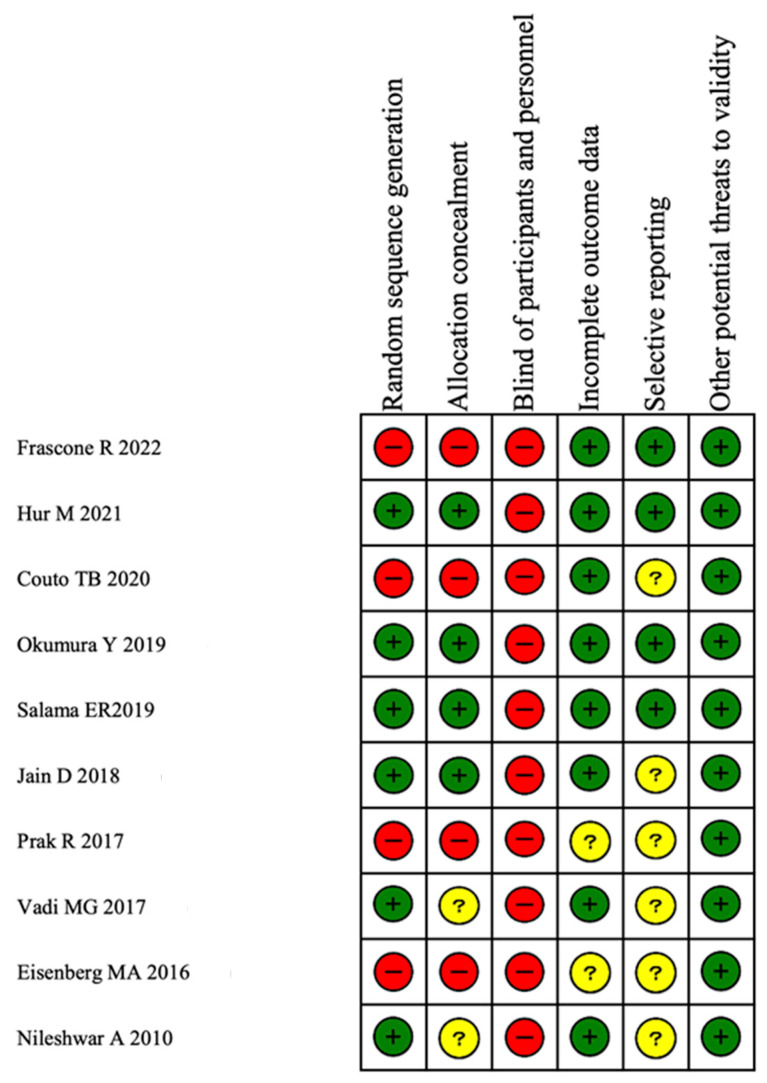
The risk of bias assessment. Green circles, red circles, and yellow circles indicate “low risk of bias”, “high risk of bias”, and “unclear risk of bias”, respectively [5,7,8,18,19,20,22,23,24].

The risk of bias is shown in Figure 6. This study included three observational studies, so bias was observed in random sequence generation and allocation concealment. Furthermore, because the type of laryngoscope used for tracheal intubation could not be blinded, bias was observed in the blinding items in all studies. Most studies did not pre-register study protocols, creating a risk of bias.

## 4. Discussion

This meta-analysis found no significant difference in intubation failure or intubation time between the indirect laryngoscope and the direct laryngoscope in the setting of a difficult pediatric airway.

An indirect laryngoscope can be used effectively in adults when intubation is difficult. Previous studies have found that the indirect laryngoscope is superior in terms of success rate in obese adults and for manual in-line stabilization [26,27]. In a previous report, Nileshwar et al. performed tracheal intubation using a Bullard laryngoscope and the Macintosh laryngoscope in pediatric patients aged 2 to 10 years with simulated restriction of cervical spine movements, and reported that the success rate was significantly improved with the Bullard laryngoscope [5]. In addition, Jain et al. used the Miller version of the CMAC video laryngoscope and a conventional Miller laryngoscope for the tracheal intubation of infants in the lateral position and reported that intubation time was significantly reduced when using the CMAC video laryngoscope [21]. Thus, indirect laryngoscopes are expected to increase the success rate and shorten intubation time during tracheal intubation compared to direct laryngoscopes. However, in this study, indirect laryngoscopy was not as useful for tracheal intubation in difficult airway patients compared with direct laryngoscopy. A reason why indirect laryngoscopes are non-advantageous for difficult airway settings in pediatrics may be that indirect laryngoscopes have not been created for pediatric patients. Most indirect laryngoscopes share the handle with adult versions, even when used in pediatrics [28,29]. Thus, when performing tracheal intubation in pediatric patients, it is necessary to replace the blade with a pediatric blade. Tracheal intubation may be difficult when using a laryngoscope with an adult handle in a pediatric patient because the handle can come into contact with the anterior chest and interfere with intubation, or balance can be lost when holding the laryngoscope [30]. Furthermore, tracheal intubation using an indirect laryngoscope is performed while watching a video screen and requires a high level of hand-eye coordination, especially when the trachea is small, as in pediatric patients [31]. In the future, the development of indirect laryngoscopes that are more specialized for pediatric patients, and as anesthesiologists become more proficient in using indirect laryngoscopes in pediatrics, will make it more advantageous to intubate pediatric patients with difficult airways. The reason why there was no significant difference in intubation time between indirect and direct laryngoscopes may be due to the relationship between successful tracheal intubation and intubation time. The intubation time included in this study is the intubation time when tracheal intubation is successful, and does not include the intubation time when tracheal intubation is unsuccessful. Therefore, if tracheal intubation fails the first time, the intubation time is not measured, but the intubation time is measured for successful intubation from the second time onward. This method of measuring intubation time is not suitable for accurately measuring intubation time generally. Other factors include the wide age range of the pediatric patients, which may have influenced the study results. This study also included a wide range of pediatric ages, from 0 to 18 years old. Simultaneous statistical analysis of research subjects, including these wide age groups, may lead to inaccurate analysis results. In the future, it may be necessary to conduct research by specifying the age group of pediatric patients. In the subgroup analysis, only the Bullard laryngoscope was significantly superior to the direct laryngoscope in terms of intubation failure, and the McGrath and Bullard laryngoscope significantly prolonged intubation time. However, these results cannot be said to be a clear conclusion because only one study was analyzed. We also analyzed the intubation failure and intubation time for pediatrics 2 or fewer years of age or research methods and found no significant difference between indirect laryngoscope and direct laryngoscope.

We also compared the rates of adverse events (desaturation, oropharyngeal injury, hoarseness, laryngospasm, and esophageal intubation) between the two types of laryngoscope when used for tracheal intubation in pediatric patients with a difficult airway. Desaturation and esophageal intubation were significantly less common with an indirect laryngoscope than with a direct laryngoscope. On the other hand, there was no significant difference in the frequency of oropharyngeal injury, hoarseness, or laryngospasm between the two types of laryngoscope.

Reducing desaturation during endotracheal intubation is highly beneficial in preventing hypoxia and the potentially fatal complications secondary to hypoxia in pediatric patients. Therefore, desaturation during tracheal intubation must be prevented. Desaturation during tracheal intubation generally occurs when the intubation time is prolonged, except in patients with a poor respiratory status. However, in this study, indirect laryngoscopy significantly reduced desaturation during tracheal intubation, but did not significantly shorten intubation time, compared with direct laryngoscopy. These results may seem contradictory at first glance, but the intubation time with indirect laryngoscopy was about 4 s shorter than with direct laryngoscopy, although there was no difference. This suggests that in this study, the reduction in intubation time with indirect laryngoscopy led to a reduction in desaturation. However, the definition of desaturation varies among the RCTs included in the present study. In this meta-analysis, we analyzed three studies on desaturation. Among them, two studies used <80% for desaturation and one study used <95% for desaturation. Inconsistent definitions of desaturation can lead to biased research results. Furthermore, our findings regarding desaturation may be inaccurate due to the small number of patients, and further research is needed.

Indirect laryngoscopy also significantly reduced esophageal intubation compared with direct laryngoscope in this analysis. Pediatric patients are anatomically smaller and their trachea and esophagus are closer together than in adults. It can be difficult to secure a visual field inside the oral cavity, and the tracheal tube may be accidentally inserted into the esophagus. Since the oral cavity is narrow, it can be difficult to insert the tracheal tube within the oral cavity, and it is difficult to guide the tracheal tube to the glottis. This can lead to esophageal intubation. A major advantage of indirect laryngoscopy is that it visualizes the upper airway, including the glottis, on an external monitor. By displaying the glottis on a monitor, the glottis can be visualized in a larger size, and the glottis can be seen closer, making it easier to differentiate between the glottis and the esophagus. Since there is no need to secure a visual field within the narrow oral cavity, it becomes easier to guide the tracheal tube to the glottis. Due to these factors, indirect laryngoscopy appears to have significantly reduced the number of esophageal intubations. However, our findings regarding esophageal intubation rates may be inaccurate because of the small number of patients. Further research is needed.

Laryngospasm is a condition in which muscle groups in the larynx cause the glottis to close and is a defensive reflex mediated by the vagus nerve [32]. Saliva, blood, vomit, instrument manipulation near the larynx, and painful stimuli can all be triggers and occur via the superior laryngeal nerve [32]. When laryngospasm occurs, the vocal cords, false vocal cords, and arytenoids are fixed in the midline. As a result, the upper airway becomes completely obstructed. If left untreated, hypoxemia will rapidly progress, resulting in myocardial ischemia, cerebral ischemia, and death. The fundamental treatment is to quickly relieve convulsions and reoxygenate the patient. There are many triggers that can cause laryngospasm during the induction of general anesthesia, and tracheal intubation is one of them. Past reports indicate that laryngospasm occurs at approximately 1% probability during anesthesia induction [33]. In this study, the incidence of laryngospasm during tracheal intubation was similar between indirect and direct laryngoscopes as is difficult to prevent laryngospasm by simply changing the type of laryngoscope due to factors that induce laryngospasm, including the inflow of saliva into the trachea, the use of inhaled anesthetics, and the depth of anesthesia.

In this study, there was no difference in the incidence of oropharyngeal injury between indirect and direct laryngoscopy. It is controversial whether the use of indirect laryngoscopy increases the incidence of oropharyngeal injury [34].

A disadvantage of indirect laryngoscopy is the potential for visual and cognitive blind spots. Operating the endotracheal tube while looking at the monitor does not allow one to recognize phenomena occurring in the blind spot outside of the monitor. Therefore, this non-visual operation may lead to complications such as intraoral damage [35]. On the other hand, indirect laryngoscopes have a lower lifting force during larynx expansion, which may reduce intraoral damage. Previous reports have shown that the lifting force of Artraq during tracheal intubation is approximately one-fifth that of Macintosh laryngoscopes (Macintosh laryngoscope; 48.8 ± 15.8 N vs. Artraq; 10.4 ± 2.8 N) [36]. Also, previous reports have shown that video laryngoscopes, such as Glidescope and C-MAC video laryngoscope, reduce lifting forces during laryngoscopy [37]. Previous meta-analyses have shown that indirect laryngoscopy is less likely to cause oropharyngeal injury in pediatric patients compared to direct laryngoscopy [38]. However, this study differs from the current study because it included patients with both normal and difficult airway conditions. A recent report from the *New England Journal of Medicine* found that, in 1417 patients, indirect laryngoscopy did not increase the incidence of oropharyngeal injury during endotracheal intubation in critically ill adult patients [25,39]. In this meta-analysis, indirect laryngoscopy did not increase the incidence of oropharyngeal injury in difficult airway conditions.

There was no difference in the incidence of hoarseness between indirect and direct laryngoscopy. The occurrence of hoarseness during tracheal intubation includes dislocation of the arytenoid cartilage and damage to the vocal cords due to intubation. Using an indirect laryngoscope allows tracheal intubation to be performed with less lifting force, thereby preventing dislocation of the arytenoid cartilage. Additionally, indirect laryngoscopy allows for close observation of the glottis, increasing the possibility of intubation without damaging the vocal cords. Therefore, indirect laryngoscopy is expected to reduce the occurrence of hoarseness. However, in this study, indirect laryngoscopy did not reduce the occurrence of hoarseness. It is thought that the causes of hoarseness include factors other than the intubation operation, such as the length of time the tracheal tube is left in place, the cough reflex, and the manipulation of the tracheal tube during extubation. Previous reports have shown no difference in the occurrence of hoarseness between indirect laryngoscopy and direct laryngoscopy [38]. In their report, they combined four randomized controlled trials and reported that indirect laryngoscopy did not reduce the incidence of hoarseness in pediatric tracheal intubation (risk ratio 1.35, 95% confidence interval 0.77–2.36). However, due to the small sample size in this study, further research is needed.

## 5. Limitations

Our study has several research limitations. First, it includes prospective studies (including randomized controlled trials) and observational studies. Analyzing prospective studies and observational studies simultaneously increases heterogeneity due to different research methods. Therefore, we analyzed prospective studies and observational studies separately, which reduced the heterogeneity of prospective studies and improved the precision of the analysis. A second limitation is that this study included a variety of indirect laryngoscopes. Each indirect laryngoscope has a different method of use. In particular, the methods for using an indirect laryngoscope with a tracheal tube guide and an indirect laryngoscope without a tracheal tube guide are very different. Uniform analysis of such non-identical laryngoscopes will result in bias. Third, the definition of difficult intubation differs in each study. The definitions of difficult intubation included in this study were manual in-line stabilization (two studies), intubation in the emergency department (three studies), torticollis, cleft lip and palate, lateral positioning, and predicted difficult intubation. These difficult intubation models have different reasons for intubation difficulty, so analyzing them simultaneously would introduce bias. A fourth limitation is the wide range of ages in the pediatric groups. The age range of the pediatrics included in this study was 0–18 years. Analyzing pediatrics aged 0 and 18 years at the same time would introduce bias.

## 6. Conclusions

In this study, the intubation failure and intubation time when using an indirect laryngoscope were the same as those when using a direct laryngoscope in pediatric patients with a difficult airway. Indirect laryngoscopes significantly reduced desaturation and esophageal intubation events compared with direct laryngoscopes. On the other hand, there was no significant difference in the frequency of oropharyngeal injury, hoarseness, or laryngospasm between the two types of laryngoscope. Currently, the benefits of indirect laryngoscopes have not been observed in the setting of difficult pediatric airways.

## Figures and Tables

**Table 1:** Patients’ characteristics.

	Author	Year	Type of Laryngoscopes	Number of Participants	Patients Age	ASA-PS	Airway Condition	Study Design
1	Frascone R [18]	2022	Macintosh	28	0–17 y	N/A	Helicopter Emergency Medical Service	Retrospective
			C-MAC	27				
2	Hur M [19]	2021	Macintosh	15	1–10 y	I–II	Torticollis	RCT
			McGrath	15				
3	Couto TB [7]	2020	Macintosh	141	1–19 y	N/A	Emergency department	Prospective
			McGrath	50				
4	Okumura Y [20]	2019	Macintosh	20	3–11 m	I	Cleft lip and palate	RCT
			Airway Scope	20				
5	Salama ER [24]	2019	Miller	30	<2 y	I–II	Lateral position	RCT
			Glidescope	30				
6	Jain D [21]	2018	Miller	32	<1 y	I–III	Lateral position	RCT
			C-MAC	31				
7	Prak R [8]	2017	Macintosh	140	8.1 (1.9–12.8) or 22.5 (11.8–35.3)	N/A	Predict difficult intubation	Retrospective
			C-MAC	370				
8	Vadi MG [22]	2017	Miller	31	2 m–24 m	I–III	Manual in-line stabilization	RCT
			Glidescope	31				
			C-MAC (Storz)	31				
9	Eisenberg MA [23]	2016	Macintosh or Miller	240	0–18 y	N/A	Emergency department	Retrospective
			C-MAC	199				
10	Nileshwar A [5]	2010	Macintosh	20	2–10 y	I–II	Manual in-line stabilization	RCT
			Bullard	20				

y: year, m: month, N/A: not available, ASA-PS: American Society of Anesthesiologists Physical Status, RCT: randomized controlled trial.

## Data Availability

Publicly available datasets were analyzed in this study.

## Supplementary Materials

The following supporting information can be downloaded at: https://www.mdpi.com/article/10.3390/children11010060/s1, Supplemental S1: The methods of risks of bias assessment. Supplemental S2: The methods of Grading of Recommendations Assessment, Development, and Evaluation (GRADE) approach. Supplemental S3: Forest plot comparing the intubation failure for individual indirect and direct laryngoscopes. Supplemental S4: Forest plot comparing the intubation time for individual indirect and direct laryngoscopes. Supplemental S5: Forest plot comparing the intubation failure between indirect and direct laryngoscopes for patients under 2 years or less. Supplemental S6: Forest plot comparing the intubation time between indirect and direct laryngoscopes for patients under 2 years or less. Supplemental S7: Forest plot comparing the intubation failure for indirect and direct laryngoscopes in prospective and retrospective studies.
