# Supplementary material for: Comparison of Indirect and Direct Laryngoscopes in Pediatric Patients with a Difficult Airway: A Systematic Review and Meta-Analysis
Source: Children (Basel). 2023 Dec 31;11(1):60. doi: 10.3390/children11010060 (PMC10814718; doi:10.3390/children11010060)

Supplemental File

Supplemental S1. The risks of bias was estimated in the following methodological domains: sequence generation; allocation concealment; blinding of participants; incomplete outcome data; selective outcome reporting; and other potential threats to validity.

Supplemental S2. The Grading of Recommendations Assessment, Development and Evaluation (GRADE) approach was applied with GRADEpro software (version 3.6 for Windows; available from http://ims.cochrane.org/revman/gradepro) to assess the quality of evidence of the main outcomes. Furthermore, the quality of evidence was based on the presence or absence of the following variables: limitations of the study design, inconsistency, indirectness, imprecision of the results and publication bias. The quality of evidence for the main outcomes was graded as very low, low, moderate or high.

Supplemental S3. Forest plot comparing the intubation failure for individual indirect and direct laryngoscopes.

	Indirect laryngoscope		Direct laryngoscope			Risk Ratio
**Study or Subgroup**	**Events**	**Total**	**Events**	**Total**	**Weight**	**M-H, Random, 95% CI**
**1.1.1 AirwayScope**						
Okumura Y 2019	0	20	0	20		Not estimable
**Subtotal (95% CI)**		**20**		**20**		**Not estimable**
Total events	0		0			
Heterogeneity: Not applicable						
Test for overall effect: Not applicable						
**1.1.2 Bullard**						
Nileshwar A 2010	9	20	0	20	3.0%	19.00 [1.18, 305.88]
**Subtotal (95% CI)**		**20**		**20**	**3.0%**	**19.00 [1.18, 305.88]**
Total events	9		0			
Heterogeneity: Not applicable						
Test for overall effect: Z = 2.08 (P = 0.04)						
**1.1.3 C-MAC**						
Prak R 2017	126	370	117	140	19.4%	0.41 [0.35, 0.48]
Jain D 2018	2	31	5	32	7.1%	0.41 [0.09, 1.97]
Eisenberg MA 2016	55	199	70	240	18.5%	0.95 [0.70, 1.28]
Frascone R 2022	8	27	5	28	11.6%	1.66 [0.62, 4.44]
Vadi MG 2017	7	31	1	15	5.1%	3.39 [0.46, 25.09]
**Subtotal (95% CI)**		**658**		**455**	**61.6%**	**0.83 [0.41, 1.69]**
Total events	198		198			
Heterogeneity: $Tau^2 = 0.43$; $Chi^2 = 37.59$, df = 4 (P < 0.00001); $I^2 = 89\%$						
Test for overall effect: Z = 0.52 (P = 0.61)						
**1.1.4 Glidescope**						
Salama ER 2019	3	30	6	30	8.9%	0.50 [0.14, 1.82]
Vadi MG 2017	5	31	1	15	4.9%	2.42 [0.31, 18.92]
**Subtotal (95% CI)**		**61**		**45**	**13.8%**	**0.89 [0.20, 4.01]**
Total events	8		7			
Heterogeneity: $Tau^2 = 0.49$; $Chi^2 = 1.64$, df = 1 (P = 0.20); $I^2 = 39\%$						
Test for overall effect: Z = 0.15 (P = 0.88)						
**1.1.5 McGrath**						
Couto TB 2020	16	50	88	141	17.4%	0.51 [0.34, 0.78]
Hur M 2021	2	15	1	15	4.1%	2.00 [0.20, 19.78]
**Subtotal (95% CI)**		**65**		**156**	**21.6%**	**0.62 [0.24, 1.60]**
Total events	18		89			
Heterogeneity: $Tau^2 = 0.22$; $Chi^2 = 1.32$, df = 1 (P = 0.25); $I^2 = 24\%$						
Test for overall effect: Z = 0.98 (P = 0.33)						
**Total (95% CI)**		**824**		**696**	**100.0%**	**0.86 [0.51, 1.46]**
Total events	233		294			
Heterogeneity: $Tau^2 = 0.36$; $Chi^2 = 50.46$, df = 9 (P < 0.00001); $I^2 = 82\%$						
Test for overall effect: Z = 0.55 (P = 0.59)						
Test for subgroup differences: $Chi^2 = 5.22$, df = 3 (P = 0.16), $I^2 = 42.6\%$						

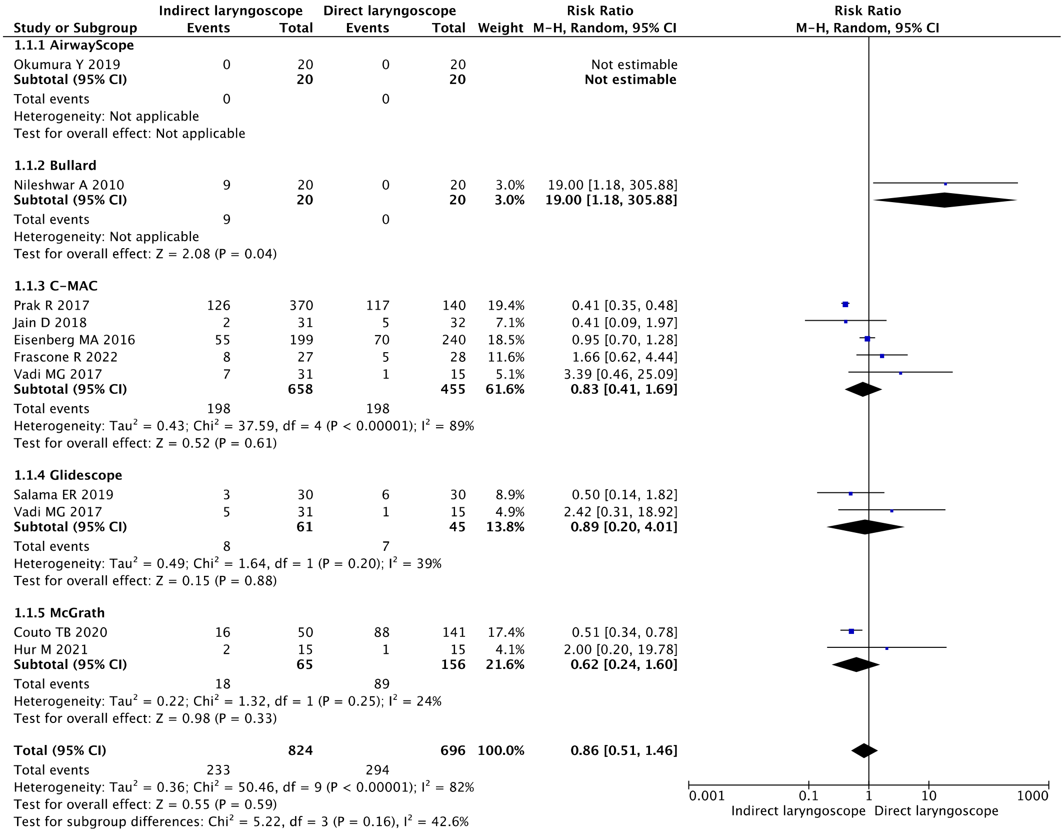

Supplemental S4. Forest plot comparing the intubation time for individual indirect and direct laryngoscopes.

	Indirect laryngoscope			Direct laryngoscope				Mean Difference
**Study or Subgroup**	**Mean**	**SD**	**Total**	**Mean**	**SD**	**Total**	**Weight**	**IV, Random, 95% CI**
**1.1.1 AirwayScope**								
Okumura Y 2019	31.5	8.3	20	26.4	8.9	20	15.4%	5.10 [−0.23, 10.43]
**Subtotal (95% CI)**			**20**			**20**	**15.4%**	**5.10 [−0.23, 10.43]**
Heterogeneity: Not applicable								
Test for overall effect: Z = 1.87 (P = 0.06)								
**1.1.2 Bullard**								
Nileshwar A 2010	75.7	28.5	20	38.2	38.2	20	4.7%	37.50 [16.61, 58.39]
**Subtotal (95% CI)**			**20**			**20**	**4.7%**	**37.50 [16.61, 58.39]**
Heterogeneity: Not applicable								
Test for overall effect: Z = 3.52 (P = 0.0004)								
**1.1.3 C-MAC**								
Jain D 2018	32.1	5.5	31	38.2	10.7	32	16.4%	−6.10 [−10.28, −1.92]
Vadi MG 2017	33.3	12.7	31	23.3	4.3	15	15.8%	10.00 [5.03, 14.97]
**Subtotal (95% CI)**			**62**			**47**	**32.2%**	**1.89 [−13.89, 17.67]**
Heterogeneity: Tau^2 = 124.11; Chi^2 = 23.59, df = 1 (P < 0.00001); I^2 = 96%								
Test for overall effect: Z = 0.23 (P = 0.81)								
**1.1.4 Glidescope**								
Salama ER 2019	16.8	11.2	30	17.5	11.9	30	14.9%	−0.70 [−6.55, 5.15]
Vadi MG 2017	24.4	10.2	31	23.3	4.3	15	16.4%	1.10 [−3.10, 5.30]
**Subtotal (95% CI)**			**61**			**45**	**31.4%**	**0.49 [−2.92, 3.90]**
Heterogeneity: Tau^2 = 0.00; Chi^2 = 0.24, df = 1 (P = 0.62); I^2 = 0%								
Test for overall effect: Z = 0.28 (P = 0.78)								
**1.1.5 McGrath**								
Hur M 2021	31.4	6.7	15	26.1	5.4	15	16.3%	5.30 [0.95, 9.65]
**Subtotal (95% CI)**			**15**			**15**	**16.3%**	**5.30 [0.95, 9.65]**
Heterogeneity: Not applicable								
Test for overall effect: Z = 2.39 (P = 0.02)								
**Total (95% CI)**			**178**			**147**	**100.0%**	**4.06 [−1.18, 9.30]**
Heterogeneity: Tau^2 = 38.91; Chi^2 = 39.82, df = 6 (P < 0.00001); I^2 = 85%								
Test for overall effect: Z = 1.52 (P = 0.13)								
Test for subgroup differences: Chi^2 = 14.19, df = 4 (P = 0.007), I^2 = 71.8%								

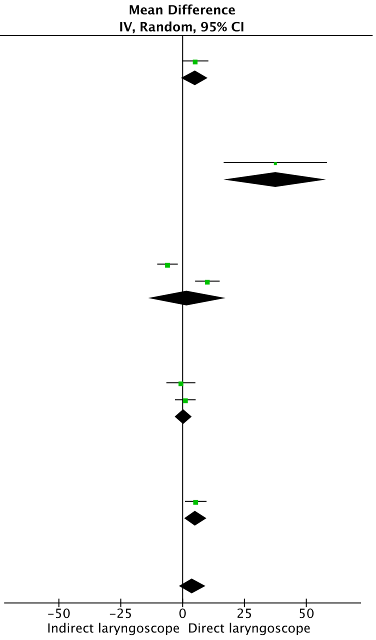

Supplemental S5. Forest plot comparing the intubation failure between indirect and direct laryngoscopes for patients under 2 years or less.

	Indirect laryngoscope		Direct laryngoscope			Risk Ratio
**Study or Subgroup**	**Events**	**Total**	**Events**	**Total**	**Weight**	**M-H, Random, 95% CI**
**1.1.2 Intubation failure 2 years or less**						
Okumura Y 2019	0	20	0	20		Not estimable
Jain D 2018	2	31	5	32	27.4%	0.41 [0.09, 1.97]
Salama ER 2019	3	30	6	30	34.7%	0.50 [0.14, 1.82]
Vadi MG 2017	5	31	1	15	18.6%	2.42 [0.31, 18.92]
Vadi MG 2017	7	31	1	15	19.3%	3.39 [0.46, 25.09]
**Subtotal (95% CI)**		**143**		**112**	**100.0%**	**0.92 [0.33, 2.53]**
Total events	17		13			
Heterogeneity: $Tau^2 = 0.34$; $Chi^2 = 4.37$, df = 3 (P = 0.22); $I^2 = 31\%$						
Test for overall effect: Z = 0.16 (P = 0.87)						
**Total (95% CI)**		**143**		**112**	**100.0%**	**0.92 [0.33, 2.53]**
Total events	17		13			
Heterogeneity: $Tau^2 = 0.34$; $Chi^2 = 4.37$, df = 3 (P = 0.22); $I^2 = 31\%$						
Test for overall effect: Z = 0.16 (P = 0.87)						
Test for subgroup differences: Not applicable						

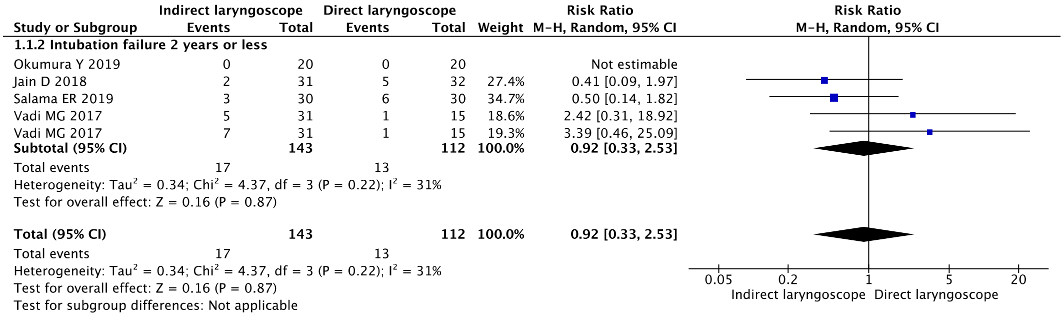

Supplemental S6. Forest plot comparing the intubation time between indirect and direct laryngoscopes for patients under 2 years or less.

	Indirect laryngoscope			Direct laryngoscope				Mean Difference
**Study or Subgroup**	**Mean**	**SD**	**Total**	**Mean**	**SD**	**Total**	**Weight**	**IV, Random, 95% CI**
**1.1.2 Intubation time 2 years or less**								
Jain D 2018	32.1	5.5	31	38.2	10.7	32	20.9%	−6.10 [−10.28, −1.92]
Salama ER 2019	16.8	11.2	30	17.5	11.9	30	18.8%	−0.70 [−6.55, 5.15]
Vadi MG 2017	24.4	10.2	31	23.3	4.3	15	20.9%	1.10 [−3.10, 5.30]
Okumura Y 2019	31.5	8.3	20	26.4	8.9	20	19.5%	5.10 [−0.23, 10.43]
Vadi MG 2017	33.3	12.7	31	23.3	4.3	15	19.9%	10.00 [5.03, 14.97]
**Subtotal (95% CI)**			**143**			**112**	**100.0%**	**1.81 [−3.73, 7.34]**
Heterogeneity: $Tau^2 = 33.58$; $Chi^2 = 26.19$, df = 4 ($P < 0.0001$); $I^2 = 85\%$								
Test for overall effect: Z = 0.64 (P = 0.52)								
**Total (95% CI)**			**143**			**112**	**100.0%**	**1.81 [−3.73, 7.34]**
Heterogeneity: $Tau^2 = 33.58$; $Chi^2 = 26.19$, df = 4 ($P < 0.0001$); $I^2 = 85\%$								
Test for overall effect: Z = 0.64 (P = 0.52)								
Test for subgroup differences: Not applicable								

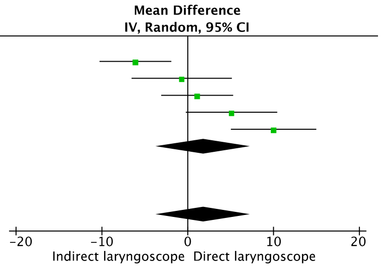

Supplemental S7. Forest plot comparing the intubation failure for indirect and direct laryngoscopes in prospective and retrospective studies.

	Indirect laryngoscope		Direct laryngoscope			Risk Ratio
**Study or Subgroup**	**Events**	**Total**	**Events**	**Total**	**Weight**	**M-H, Random, 95% CI**
**1.1.1 Prospective**						
Okumura Y 2019	0	20	0	20		Not estimable
Jain D 2018	2	31	5	32	7.1%	0.41 [0.09, 1.97]
Salama ER 2019	3	30	6	30	8.9%	0.50 [0.14, 1.82]
Couto TB 2020	16	50	88	141	17.4%	0.51 [0.34, 0.78]
Hur M 2021	2	15	1	15	4.1%	2.00 [0.20, 19.78]
Vadi MG 2017	5	31	1	15	4.9%	2.42 [0.31, 18.92]
Vadi MG 2017	7	31	1	15	5.1%	3.39 [0.46, 25.09]
Nileshwar A 2010	9	20	0	20	3.0%	19.00 [1.18, 305.88]
**Subtotal (95% CI)**		**228**		**288**	**50.5%**	**1.08 [0.45, 2.58]**
Total events	44		102			
Heterogeneity: $Tau^2 = 0.66$; $Chi^2 = 13.87$, df = 6 (P = 0.03); $I^2 = 57\%$						
Test for overall effect: Z = 0.18 (P = 0.86)						
**1.1.2 Retrospective**						
Prak R 2017	126	370	117	140	19.4%	0.41 [0.35, 0.48]
Eisenberg MA 2016	55	199	70	240	18.5%	0.95 [0.70, 1.28]
Frascone R 2022	8	27	5	28	11.6%	1.66 [0.62, 4.44]
**Subtotal (95% CI)**		**596**		**408**	**49.5%**	**0.79 [0.35, 1.75]**
Total events	189		192			
Heterogeneity: $Tau^2 = 0.43$; $Chi^2 = 33.24$, df = 2 (P < 0.00001); $I^2 = 94\%$						
Test for overall effect: Z = 0.59 (P = 0.55)						
**Total (95% CI)**		**824**		**696**	**100.0%**	**0.86 [0.51, 1.46]**
Total events	233		294			
Heterogeneity: $Tau^2 = 0.36$; $Chi^2 = 50.46$, df = 9 (P < 0.00001); $I^2 = 82\%$						
Test for overall effect: Z = 0.55 (P = 0.59)						
Test for subgroup differences: $Chi^2 = 0.28$, df = 1 (P = 0.59), $I^2 = 0\%$						

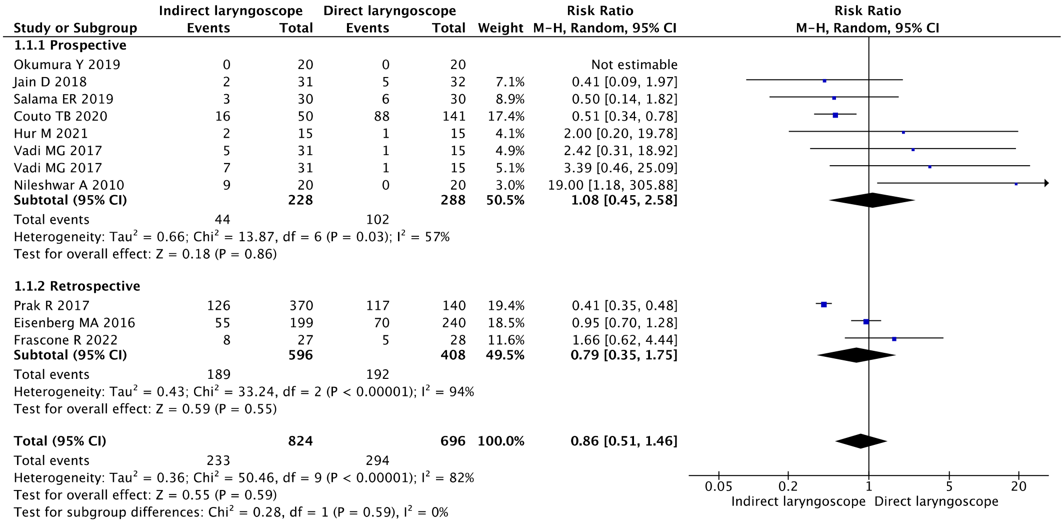

Supplement: Supplementary file 1 [file children-11-00060-s001.zip › children-2786978-supplementary.pdf]
